# A Novel Bi_2_MoO_6_/ZIF-8 Composite for Enhanced Visible Light Photocatalytic Activity

**DOI:** 10.3390/nano9040545

**Published:** 2019-04-04

**Authors:** Yu Xia, Shao-ke Shang, Xie-rong Zeng, Ji Zhou, Ya-yun Li

**Affiliations:** 1 Shenzhen Key Laboratory of Special Functional Materials & Shenzhen Engineering Laboratory for Advance Technology of Ceramics, College of Materials Science and Engineering, Shenzhen University, Shenzhen 518060, China; 2172341411@email.szu.edu.cn (Y.X.); 2172341466@email.szu.edu.cn (S.-k.S.); zengxier@szu.edu.cn (X.-r.Z.); 2 State Key Laboratory of New Ceramics and Fine Processing, School of Materials Science and Engineering, Tsinghua University, Beijing 100084, China; zhouji@tsinghua.edu.cn

**Keywords:** Bi_2_MoO_6_, ZIF-8, photocatalysis, morphology

## Abstract

A series of novel Bi_2_MoO_6_/zeolitic imidazolate framework-8 (ZIF-8) photocatalysts have been successfully fabricated through a facile self-assembly process. X-ray diffraction (XRD), scanning electron microscopy (SEM), UV-vis spectrophotometry, and X-ray photoelectron spectroscopy (XPS) characterized pure Bi_2_MoO_6_, pure ZIF-8, and a series of Bi_2_MoO_6_/ZIF-8 composites. The result indicated that, when compared with pure Bi_2_MoO_6_, the composite of Bi_2_MoO_6_/ZIF-8 exhibited excellent photocatalytic performance for the degradation of methylene blue (MB) under visible light. Moreover, the Bi_2_MoO_6_/ZIF-8-3 composite (the molar ratio of Bi_2_MoO_6_ to 2-MI is 3:3) has optimum photocatalytic performance because of the suitable amount of ZIF-8 decorated on the flower-like Bi_2_MoO_6_. The enhanced photocatalytic activity is probably due to the introduction of ZIF-8, which will promote the separation of electron–hole pair and the surface morphology. Benefitting from the diversity of the MOF species (ZIF-8 is one of them), this composing strategy of Bi_2_MoO_6_/MOF composite would provide new insight into the design of highly efficient visible light photocatalysts.

## 1. Introduction

Photocatalytic materials, especially semiconductor photocatalysts, have attracted a lot of attention in recent years due to the world-wide environmental problems, such as the water pollution [1,2,3]. However, as most semiconductor photocatalysts, such as TiO_2_, cannot utilize visible light and respond to other visible lights, except ultraviolet light, because of a wide band gap, the photocatalytic activity of them was reduced [4]. Therefore, for the effective use of visible lights, the semiconductor photocatalyst should be able to respond to all visible lights and its band gap should be within a suitable range.

Bismuth molybdate (Bi_2_MoO_6_) is a typical Aurivillius oxide. When compared with the titanium dioxide, the Bi_2_MoO_6_ has a smaller forbidden band width (about 2.7 eV). As a result, it has a better photocatalytic activity within the visible range [5]. As is known to all, the morphology of the semiconductor photocatalyst plays a vital role in photocatalysis process [6]. Bi_2_MoO_6_ has a variety of morphologies that have been synthesized [7,8], among which the hierarchical flower-like Bi_2_MoO_6_ spheres have attracted a lot of attention with their super photocatalytic performance. In addition, the catalytic efficiency of the semiconductor photocatalyst can be greatly improved by increasing the number of photogenerated electron-hole pairs, inhibiting the recombination of photogenerated electron-hole pairs and increasing the reaction sites of the catalyst with the reactants. The photocatalytic activity of the Bi_2_MoO_6_ photogenerated electron-hole pairing ratio is relatively high. To further improve the photocatalytic activity of Bi_2_MoO_6_, many feasible studies have been conducted, such as ion doping [9], surface noble metal deposition [10], and compounding of semiconductor photocatalysts [11]. However, there is still much room for the exploration of the potential of bismuth molybdate-based as a photocatalyst material.

Metal-organic frameworks (MOFs) is a kind of porous crystalline material. It has a periodic multi-dimensional network structure that is formed by the coordination of metal ions or metal cluster units and the self-assemble of organic ligand [12,13]. As the metals and organic ligands can be adjusted and controlled, MOFs exhibit structural diversity and have recently attracted wide attention from the public. Researchers have found that MOFs have potential to be applied in many fields, such as gas storage and separation, catalysis, sensors, drug delivery, etc. [14,15,16,17]. In addition, zeolitic imidazolate framework-8 (ZIF-8) is one of the MOFs with outstanding photocatalytic properties, which has been researched many times in the field of photocatalysis. For example, X. Zeng et al. synthesized the TiO_2_/ZIF-8 composite, which effectively inhibits the recombination of charge carrier, resulting in an improved photocatalytic activity [18]. Y.H. Ding et al. reported that Bi_2_S_3_@ZIF-8 core–shell heterostructures exhibited photocatalytic activity to Rhodamine B (RhB) dye degradation under visible light [19]. These results indicated that ZIF-8 based materials have potential applications in the field of photocatalysis.

In this work, a novel Bi_2_MoO_6_/ZIF-8 composite that displays excellent photocatalytic activity in the degradation of methylene blue (MB) has been successfully synthesized. The nanoparticle of ZIF-8, with different content, has been decorated on the hierarchical flower-like Bi_2_MoO_6_. The result shows that the Bi_2_MoO_6_/ZIF-8 composite has excellent photocatalytic performance under visible light.

## 2. Experimental Section

### 2.1. Preparation of Bi_2_MoO_6_

All of the chemicals were of analytical grade and they can be used without further purification. Bi_2_MoO_6_ powder was prepared by the hydrothermal method [20], Na_2_MoO_4_·2H_2_O (1 mmol, 0.4210 g) and Bi(NO_3_)_3_·5H_2_O (2 mmol, 1.6866 g) was dissolved in 5 mL ethylene glycol (EG), respectively. After being stirred for 10 min, the above two solutions were mixed together. Subsequently, 20 mL ethanol was added into the above mixture and then stirred for 10 min. Afterwards, the mixture was transferred into a Teflon lined steel autoclave and heated at 160 °C for 20 h. After the autoclave was cooled to room temperature, the yellow products was recovered by filter, washed with deionized water and ethanol, and collected under vacuum at 80 °C for 12 h.

### 2.2. Preparation of Bi_2_MoO_6_/ZIF-8 Composites

**Figure 1:**
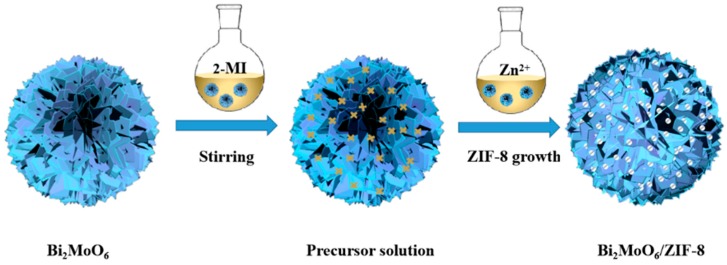
The preparation process of Bi_2_MoO_6_/ZIF-8 via the self-assembly method.

Figure 1 illustrates the preparation procedure for the Bi_2_MoO_6_/ZIF-8 composites. In general, the as-synthesized flower-like Bi_2_MoO_6_ spheres were dispersed into a methanol solution containing 2-methylimidazole (2.4 mmol, 0.66 g), and then stirred for 10 min. Zn(NO_3_)_2_·6H_2_O (0.3 mmol 0.03 g) was dispersed into 10 mL methanol. After being stirred for 10 min, the above two solutions were mixed together and stirred for 10 min. The samples were collected by centrifugation (7000 rpm, 5 min), washed by methanol for three times, and finally the Bi_2_MoO_6_/ZIF-8 composites were dried at 80 °C for 12 h. All of the Bi_2_MoO_6_/ZIF-8 composites with different molar ratios of Bi_2_MoO_6_ to 2-methylimidazole (2-MI), i.e., 5:3, 4:3, 3:3, and 2:3, respectively, were also prepared under the similar procedure. The samples were named Bi_2_MoO_6_/ZIF-8-1, Bi_2_MoO_6_/ZIF-8-2, Bi_2_MoO_6_/ZIF-8-3, and Bi_2_MoO_6_/ZIF-8-4, respectively according to the different molar ratio of Bi_2_MoO_6_ to 2-MI mentioned above. The syntheses of pure ZIF-8 without Bi_2_MoO_6_ were prepared in the same way.

### 2.3. Characterization

X-ray powder diffraction (XRD) patterns were obtained from a Karlsruhe D8 Advance powder diffractometer (Bruker, Karlsruhe, Germany) with Cu Kα X-ray radiation (k = 0.15418 nm). A scanning electron microscope (Hitachi SU70, Tokyo, Japan) characterized the morphology images of the Bi_2_MoO_6_/ZIF-8 composite. Chemical state were analyzed by using X-ray photoelectron spectroscopy (Escalab 250XI, Thermo Fisher Scientific, Waltham, MA, USA).

### 2.4. Evaluation of Photocatalytic Performance

In this experiment, 20 mg photocatalysts were added to 80 mL aqueous solution of Methylene blue (20 mg/L). To ensure an adsorption/desorption equilibrium, the solution was continuously stirred for 30 min in the dark before light irradiation. Afterwards, turn on the light source, which is made up of 300 W Xe lamps with 420 nm cut-off filters. Take about 4 mL of suspension every 20 min and centrifuge it at the same time. The degradation of methylene blue solution and the UV-visible absorption/diffuse reflectance spectroscopy were characterized by a UV-2450 spectrometer (Shimadzu, Kyoto, Japan).

## 3. Results and Discussion

### 3.1. XRD Analysis

**Figure 2:**
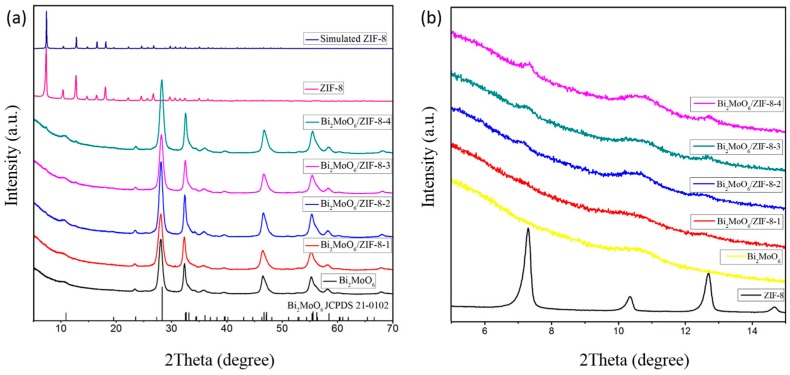
X-ray powder diffraction (XRD) Patterns of simulated zeolitic imidazolate framework-8 (ZIF-8), pure ZIF-8, Bi_2_MoO_6_, Bi_2_MoO_6_/ZIF-8-1, Bi_2_MoO_6_/ZIF-8-2, Bi_2_MoO_6_/ZIF-8-3, Bi_2_MoO_6_/ZIF-8-4 (**a**); the magnified XRD pattern of pure ZIF-8, Bi_2_MoO_6_, Bi_2_MoO_6_/ZIF-8-1, Bi_2_MoO_6_/ZIF-8-2, Bi_2_MoO_6_/ZIF-8-3, Bi_2_MoO_6_/ZIF-8-4 (**b**).

Figure 2a shows the X-ray diffraction pattern of simulated ZIF-8, synthesized pure ZIF-8, pure Bi_2_MoO_6_, and a series of the composite Bi_2_MoO_6_/ZIF-8-1, Bi_2_MoO_6_/ZIF-8-2, Bi_2_MoO_6_/ZIF-8-3, and Bi_2_MoO_6_/ZIF-8-4. The diffraction peak of the synthesized ZIF-8 conforms to that of the simulated ZIF-8. It indicates that the synthesized samples are pure. The diffraction peak of synthesized Bi_2_MoO_6_ can be indexed as Bi_2_MoO_6_ (JCPDS No. 21-0102). Moreover, in Figure 2b, with the increased content of ZIF-8 in the series of composites of Bi_2_MoO_6_/ZIF-8-(1 to 4), the intensity of the ZIF-8 peak will also be enhanced, but it is not obvious. It is probably because of the low content and crystallinity of ZIF-8 [21]. Subsequent characterization results further support the successful combination of ZIF-8 and Bi_2_MoO_6_.

### 3.2. Scanning Electron Microscopy (SEM) Analysis

**Figure 3:**
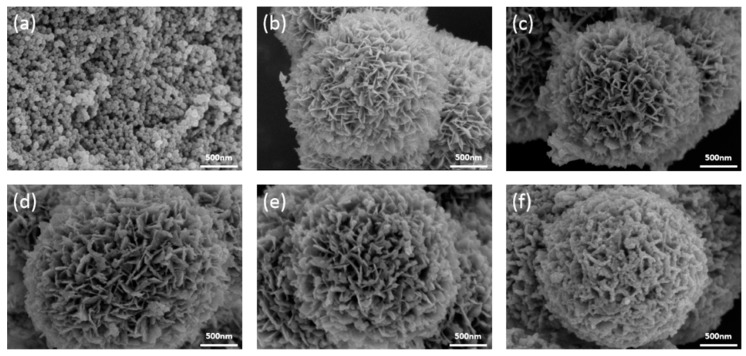
Scanning Electron Microscopy (SEM) image of pure ZIF-8 (**a**), pure Bi_2_MoO_6_ (**b**), Bi_2_MoO_6_/ZIF-8-1 (**c**), Bi_2_MoO_6_/ZIF-8-2 (**d**), Bi_2_MoO_6_/ZIF-8-3 (**e**), and Bi_2_MoO_6_/ZIF-8-4 (**f**).

**Figure 4:**
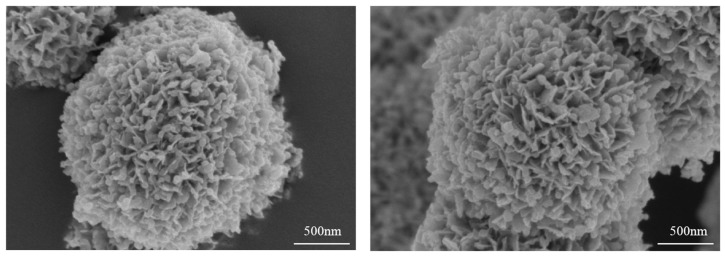
SEM image of Bi_2_MoO_6_/ZIF-8-3 in different positions.

As can be clearly observed in Figure 3a, the SEM image of pure ZIF-8 exhibits regular nanoparticle morphology, with average length about 50 nm. As shown in Figure 3b, the hierarchical flower-like Bi_2_MoO_6_ spheres with diameter of about 2 μm are composed of many smooth and clean nanosheets. Figure 3c–f shows the morphologies of Bi_2_MoO_6_/ZIF-8-1, Bi_2_MoO_6_/ZIF-8-2, Bi_2_MoO_6_/ZIF-8-3 and Bi_2_MoO_6_/ZIF-8-4. The amount of ZIF-8 is too small to be observed in Figure 3c. It is obvious that a combination of different content of ZIF-8 is formed on the nanosheets of Bi_2_MoO_6_ in Figure 3d–f. As the content of ZIF-8 increases, the amount of ZIF-8 on the sheet of Bi_2_MoO_6_ increases. When the molar ratio of Bi_2_MoO_6_ to 2-MI is 2:3, Bi_2_MoO_6_ is wrapped by ZIF-8 and the photocatalytic performance of Bi_2_MoO_6_/ZIF-8 will also be affected. Figure 4 shows more SEM images of Bi_2_MoO_6_/ZIF-8-3, which will further demonstrate that the proper amount of ZIF-8 were successfully combined on the Bi_2_MoO_6_ with an optimal morphology.

### 3.3. X-ray Photoelectron Spectroscopy (XPS) Analysis

**Figure 5:**
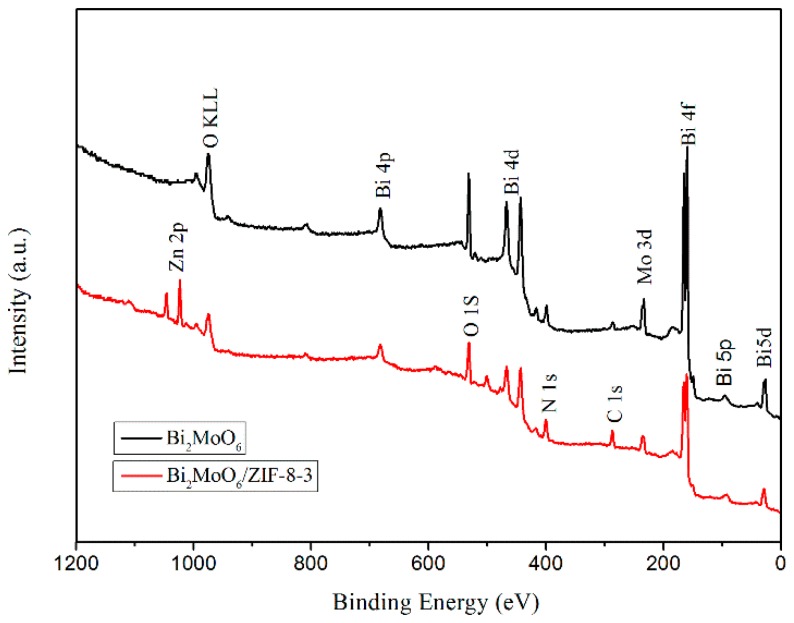
Survey X-ray photoelectron spectroscopy (XPS) spectrum of pure Bi_2_MoO_6_ and Bi_2_MoO_6_/ZIF-8-3.

**Figure 6:**
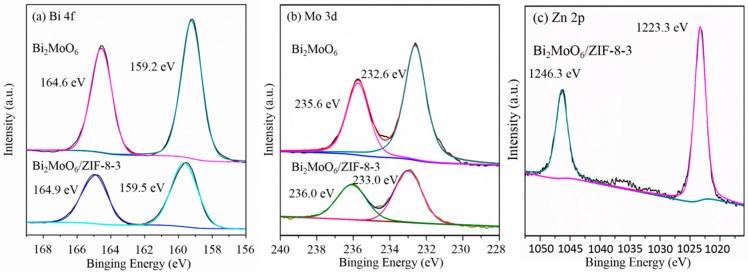
Survey XPS spectrum of pure Bi_2_MoO_6_ and Bi_2_MoO_6_/ZIF-8-3, high-resolution spectrum of Bi 4f (**a**), Mo 3d (**b**), and Zn 2p (**c**).

The X-ray photoelectron spectroscopy spectrum demonstrates the chemical compositions and the oxidation state of pure Bi_2_MoO_6_ and Bi_2_MoO_6_/ZIF-8-3. From the full survey spectrum in Figure 5, it is obvious that a Bi, Mo, O, and C element exits in the sample of pure Bi_2_MoO_6_. In addition, when compared with pure Bi_2_MoO_6_, the composite of Bi_2_MoO_6_/ZIF-8 has two more elements, i.e., Zn and N. In Figure 6a, for the pure Bi_2_MoO_6_, the two peaks centered at 159.2 eV and 164.6 eV can be ascribed to the Bi 4f_7/2_ and Bi 4f_5/2_ of Bi^3+^ [22]. In addition, the peaks of Bi_2_MoO_6_/ZIF-8 composite showed a tiny shift when compared with pure Bi_2_MoO_6_. In Figure 6b, the two peaks that are centered at 232.6 eV and 235.6 eV correspond to the Mo 3d_5/2_ and Mo 3d_3/2_ binding energies of Mo^6+^ [23]. For the composites of Bi_2_MoO_6_/ZIF-8, the binding energy of Mo 3d_5/2_ and Mo 3d_3/2_ increase 0.4 eV. In Figure 6c, the two peaks that were centered at 1023.3 eV and 1046.2 eV were attributed to the Zn 2p_3/2_ and Zn 2p_1/2_ of Zn^2+^ in the composites of Bi_2_MoO_6_/ZIF-8 [24]. These results showed that ZIF-8 has been successfully decorated in Bi_2_MoO_6_ spheres.

### 3.4. Photocatalytic Activities

**Figure 7:**
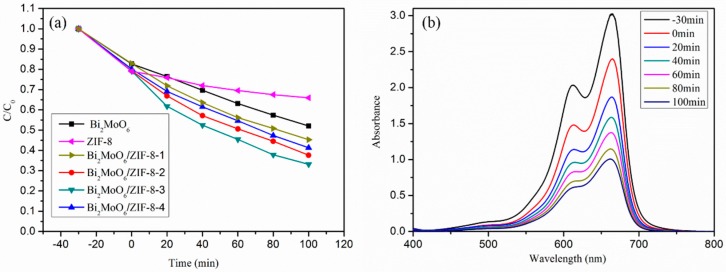
The photocatalytic degradation efficiencies of different photocatalysts to degrade methylene blue (MB) (**a**); Time dependent UV–vis spectrum changes of MB catalyzed by Bi_2_MoO_6_/ZIF-8-3 (**b**).

As shown in Figure 7a, the degradation of MB under the visible light irradiation determined the photocatalytic degradation activities of different photocatalysts. The MB photodegradation rate of pure Bi_2_MoO_6_ is 47.89% and that of pure ZIF-8 is 34.05%. When the composite is Bi_2_MoO_6_/ZIF-8-3, the degradation of MB increased to 66.88%. From the time dependent UV–vis spectrum changes of MB catalyzed by Bi_2_MoO_6_/ZIF-8-3, as shown in Figure 7b, it is obvious that it has excellent adsorption performance and photocatalytic performance. In addition, the photocatalytic performance of the composite of Bi_2_MoO_6_/ZIF-8 is improved when compared with that of the pure Bi_2_MoO_6_. Therefore, ZIF-8 plays an important role in the degradation process. In the first three groups of composites (Bi_2_MoO_6_/ZIF-8-(1 to 3)), the photocatalytic performance of the composite gradually increases with the increase of photocatalysts content. Continuing to increase the content of ZIF-8 will reduce its performance, which is possibly due to the fact that the generation of exceeding ZIF-8 on the sheet of Bi_2_MoO_6_ inhibited its absorption of light. This phenomenon also corresponds to the results of the previous SEM.

Accordingly, in order to understand the photocatalytic mechanism of these composites, a possible mechanism for the photocatalytic degradation of MB over Bi_2_MoO_6_/ZIF-8 composites is proposed. In the Bi_2_MoO_6_/ZIF-8 composites, the conduction band (CB) and valence band (VB) of Bi_2_MoO_6_ are −0.32 eV and 2.34 eV [25] and the CB and VB of ZIF-8 are −3.41 eV and 1.68 eV [24]. Under the irradiation of the light source, Bi_2_MoO_6_ is more prone to generating the photogenerated electron-hole pair and the VB of Bi_2_MoO_6_ is more positive than that of ZIF-8, the photogenerated hole that is generated by Bi_2_MoO_6_ would transfer the VB of ZIF-8, which would promote the separation of electron-hole pairs and improve the photocatalytic performance of the composite.

## 4. Conclusions

In summary, a series of Bi_2_MoO_6_/ZIF-8 composites with a different content of ZIF-8 have been successfully fabricated by a facile self-assembly method. The photocatalysts of Bi_2_MoO_6_/ZIF-8 have better performance than pure Bi_2_MoO_6_ in the degradation of MB under the visible light. The successful composition of ZIF-8 and Bi_2_MoO_6_ inhibited the recombination of a photogenerated electron–hole pair. When the molar ratio of Bi_2_MoO_6_ to 2-MI is 3:3, the amount of ZIF-8 that is decorated on flower-like Bi_2_MoO_6_ is suitable and the ability of photocatalysis would reach the maximum. This composing strategy of Bi_2_MoO_6_/ZIF-8 would provide a new route in the designing of highly efficient visible light photocatalysts.
